# Indiana Dental College

**Published:** 1888-07

**Authors:** 


### Indiana Dental College.

The ninth annual commencement of the Indiana Dental College was held in the Plymouth Church, Indianapolis, Ind., on Wednesday evening, March 7, 1888.

The address on behalf of the Faculty was delivered by L. S. Henthorne, M.D., and the address to the class by Wm. H. Atkinson, M.D., D.D.S.

The degree of D·D.S. was conferred on the following graduates by W. L. Heiskell, D.D.S., President of the Board of Trustees :

| NAME. | RESIDENCE. | NAME. | RESIDENCE. |
|---|---|---|---|
| W. A. Alexander | Illinois. | W. J. P. Lawton | Nebraska. |
| J. L. Barnes | Illinois. | J. W. Lopp | Indiana. |
| W. H. Beeson | Indiana. | C. L. Lange | Wisconsin. |
| C. P. Curtis | Indiana. | J. S. McCurdy | Indiana, |
| R. H. Clark | Massachusetts. | R. T. Oliver | Indiana. |
| J. H. Daugherty | Indiana. | G. W. Raber | Wisconsin. |
| H. S. Hicks | Indiana. | E. Reese | Ohio. |
| W. M. Jones | Indiana. | L. A. Stewart | Indiana. |
| M. de F. Kee | Ohio. | | |

